# Measuring protein levels in planarians using western blotting

**DOI:** 10.1016/j.xpro.2020.100274

**Published:** 2021-01-14

**Authors:** Benjamin Ziman, Néstor J. Oviedo

**Affiliations:** 1 Department of Molecular & Cell Biology, University of California, Merced, CA, USA; 2 Quantitative and Systems Biology Graduate Program, University of California, Merced, CA, USA; 3 Health Sciences Research Institute, University of California, Merced, CA, USA

**Keywords:** Model organisms, Molecular biology, Protein biochemistry

## Abstract

In the planarian field, two techniques are mostly used for protein detection: immunohistochemistry (IHC) and western blotting. While IHC is great for visualizing the spatial distribution of proteins in whole organisms, it has limitations in antibody availability and issues related to nonspecific expression. The use of western blotting can circumvent nonspecific expression, providing a dependable way to quantify proteins of interest. Here, we present a standardized, easily reproducible protocol with details on protein extractions of whole planarians and western blotting.

For complete details on the use and execution of this protocol, please refer to Ziman et al. (2020a).

## Highlights

• Easy and dependable way to assess protein levels in planarians
• Extraction and measurements of total proteins from entire organisms
• Alternative approach to staining with high background in whole mount

## Graphical Abstract

**Figure:**
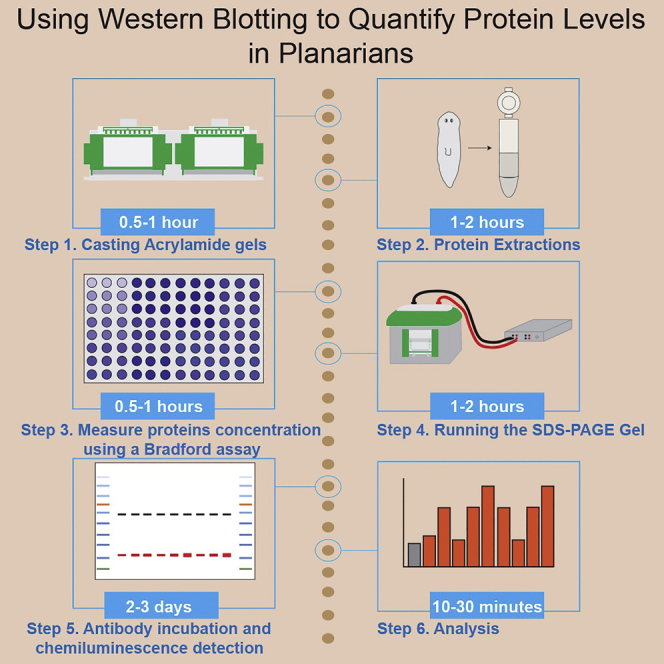

## Before you begin

### Preparation for acrylamide gels

**Timing: 5–30 min**

1. Prepare 1× Running buffer, 1 L. Refer to Materials and equipment for the buffer recipe. (Store at 4°C before use. Prepare fresh each time.)
  a. 1× Running buffer will contain 100 mL 10× Running buffer and 900 mL MilliQ water.
2. Prepare 10% ammonium persulfate (APS), 1 mL. (Store at 4°C. Prepare fresh and use within 7 days.)
  a. Dissolve 0.1 g APS into MilliQ water for a final volume of 1 mL.
3. Prepare 10% sodium dodecyl sulfate (SDS), 10 mL. (Store at 25°C. Solution will remain viable for weeks to months, no need to prepare fresh each time.)
  a. Dissolve 1 g SDS into MilliQ water for a final volume of 10 mL.
4. Prepare 1.0 M Tris pH 6.8, 50 mL. (Store at 25°C. Solution will remain viable for weeks to months, no need to prepare fresh each time.)
  a. Dissolve 6.06 g Tris base into MilliQ water, pH to 6.8 for a final volume of 50 mL.
5. Prepare 1.5 M Tris pH 8.8, 50 mL. (Store at 25°C. Solution will remain viable for weeks to months, no need to prepare fresh each time.)
  a. Dissolve 9.09 g Tris base into MilliQ water, pH to 8.8 for a final volume of 50 mL.

### Preparation for protein extractions

**Timing: 0.5–1 h**

6. Prepare protease inhibitor cocktail, 1.5 mL. (Store at −20°C, 150 μL aliquots. Solution will remain viable for weeks to months, no need to prepare fresh each time.)
  a. Dissolve 1 protease cocktail tablet into 1.5 mL of MilliQ water.
7. Prepare 100 mM phenylmethanesulfonyl fluoride (PMSF), 10 mL. (Store at −20°C, 1 mL aliquots. Solution will remain viable for weeks to months, no need to prepare fresh each time.).
  a. Dissolve 0.17 g PMSF into 10 mL of isopropanol.
8. Prepare 100 mM DL-dithiothreitol (DTT), 10 mL. (Store at −20°C, 0.5 mL aliquots. Solution will remain viable for weeks to months, no need to prepare fresh each time.).
  a. Dissolve 0.154 g DTT into 10 mL of MilliQ water.
9. Prepare 0–10 μg/μL bovine serum albumin (BSA) standards (BSA standards can also be purchased).
10. Acclimate Bradford Reagent to 25°C.
11. Collect protein extraction tools:
  a. Pellet pestle cordless motor.
  b. RNase-free disposable pellet pestles (×1/sample).
  c. 1.5 mL centrifuge tubes (×2/sample).
  d. 0.2 mL PCR tubes (×3/sample).
  e. MilliQ water.
  f. 96 well flat bottom plate.
  g. Multichannel p300 pipette.
  h. Multichannel disposable solutions basin.
  i. Centrifuge 5430R cooled to 4°C.

### Preparation of western blotting buffers

**Timing: 0.5–1 h**

12. Prepare 6× Laemmli buffer, 25 mL. Refer to Materials and equipment for the buffer recipe. (Store at −20°C, 500 μL aliquots. Solution will remain viable for weeks to months, no need to prepare fresh each time.)
13. Prepare 1× Transfer buffer, 1 L. Refer to Materials and equipment for the buffer recipe. (store at 4°C before use. Prepare fresh.)
  a. 1× Transfer buffer will contain 100 mL 10× Transfer buffer, 100 mL 100% methanol, and 800 mL MilliQ water.
14. Prepare 1× PBS, 1 L. Refer to Materials and equipment for the buffer recipe. (Store at 25°C. Solution will remain viable for weeks to months, no need to prepare fresh each time.).
  a. 1× PBS will contain 100 mL 10× PBS, pH 7.0, and 900 mL MilliQ water.
15. Prepare 1× TBST, 1 L. Refer to Materials and equipment for the buffer recipe. (Store at 25°C. Solution will remain viable for weeks to months, no need to prepare fresh each time.).
16. Prepare 1× TBST/SDS, 250 mL. Refer to Materials and equipment for the buffer recipe. (Store at 25°C. Solution will remain viable for weeks to months, no need to prepare fresh each time.).
17. Freeze the ice pack for the transfer process.

### Planarian starvation

**Timing: at least 1 week before the experiment**

18. Put a halt to feeding animals at least 1 week before beginning experiments. Make sure to clean animals at least twice during this week, by replacing water and removing mucus from their container.)

***Note:*** Animals should be starving for at least a week, as food particles may produce nonspecific antibody binding.

## Key resources table

**Table:** 

REAGENT or RESOURCE	SOURCE	IDENTIFIER
**Antibodies**		

Rabbit anti-caspase	Abcam	Cat#13847
Mouse anti-actin	Developmental Studies Hybridoma Bank	Cat#JLA20
HRP, goat anti-rabbit	Millipore Sigma	Cat#112-348
HRP, goat anti-mouse	Invitrogen	Cat#G21040

**Chemicals, peptides, and recombinant proteins**		

Acrylamide, 30%	GenDEPOT	Cat#50-101-5462
Ammonium persulfate (APS)	Bio-Rad	Cat#1610700
Tetramethylenediamine (TEMED)	Bio-Rad	Cat#1610800
Tris base	Fisher	Cat#T393-500
10× RIPA buffer	Cell Signaling Technology	Cat#9806S
cOmplete, mini, EDTA-free Protease Inhibitor Cocktail	Roche	Cat#4693159001
Phenylmethanesulfonyl fluoride (PMSF)	Millipore Sigma	Cat#10837091001
DL-Dithiothreitol (DTT)	Millipore Sigma	Cat#D9779-5G
Halt Phosphatase Inhibitor Cocktail	Thermo Scientific	Cat#1862495
Isopropanol	Fisher	Cat#A516-500
Sodium dodecyl sulfate (SDS) powder	Sigma-Aldrich	Cat#L4390
Glycerol	Acros organics	Cat#AC41098-5000
2-Mercaptoethanol	Fisher	Cat#034461-100
Bromophenol blue solution, 0.04%	Fisher	Cat#SI12-500
Glycine	VWR	Cat#97063-736
Luminata Forte Western HRP substrate, 100 mL	Millipore Sigma	Cat#WBLUF0100
Methanol (MeOH)	Fisher	Cat#A454-1
Non-fat dry milk	Walmart	Cat#9278117
PageRuler Protein Ladder	Thermo Fisher	Cat#26616
Potassium chloride	Sigma-Aldrich	Cat#P9541-500G
Potassium phosphate monobasic (KH_2_PO_4_)	Sigma-Aldrich	Cat#P5379
Sodium chloride (NaCl)	Fisher	Cat#BP358-1
Sodium phosphate sibasic (Na_2_HPO_4_)	Sigma-Aldrich	Cat#S3264
Hydrochloric acid (HCl) 12.1 M	Fisher	Cat#A144-500
Tween-20	Fisher	Cat#BP337-500
Restore PLUS western blot stripping buffer	Thermo Fisher Scientific	Cat#46430
Bovine serum albumin (BSA)	Sigma-Aldrich	Cat#A2153
Bradford Reagent	VWR	Cat#E530-1L

**Software and algorithms**		

WorkOut2.0 software	PerkinElmer	http://www.dazdaq.com/wo/wointro.htm
Image Lab 6.0.1	Bio-Rad	SOFT-LIT-170-9690-IL-SPC-V-6-1
ImageJ software	Schneider et al., 2012	https://imagej.nih.gov/ij/

**Other**		

Mini-PROTEAN Tetra cell casting module	Bio-Rad	Cat#1658015
Mini-PROTEAN Tetra vertical electrophoresis cell for mini precast gels, 4-gel	Bio-Rad	Cat#1658004
Mini-PROTEAN Tetra vertical electrophoresis cell for mini precast gels with Mini Trans-Blot module	Bio-Rad	Cat#1658030
Blot paper	Bio-Rad	Cat#1704998
0.2 μm Immuno-blot-PVDF membrane	Bio-Rad	Cat#1620177
Disposable pipette basin lids	Fisher	Cat#13-681-503
96-Well plates	Fisher	Cat#21-377-203
Pellet pestle cordless motor	Fisher	Cat#12-141-361
RNase-free disposable pellet pestles	Fisher	Cat# 12-141-364
Research PLUS variable adjustable volume pipettes: multichannel	Eppendorf	Cat# 13-690-052
1.5 mL centrifuge tubes	Fisher	Cat#05-408-129
0.2 mL PCR tube strips	Eppendorf	Cat# E0030124286
15 mL conical tubes	Fisher	Cat#07-200-886
50 mL conical tubes	Fisher	Cat#05-539-13
Transfer pipettes	Fisher	Cat#13-711-42
5 mL serological pipettes	Fisher	Cat#13-678-11D
Easypet3	Eppendorf	Cat#12-654-105
Victor3 1420 microplate reader	PerkinElmer	Model# Victor3 1420
Western blot incubation boxes	LI-COR	Cat#929-97201
ChemiDoc Imaging System	Bio-Rad	Cat#17001401
Blot/UV/stain-free sample tray for ChemiDoc	Bio-Rad	Cat#12003028
Centrifuge 5430R	Eppendorf	Cat#EP022620572
Thermomixer R	Eppendorf	Cat#EP022670107
Electrophoresis power supply	Fisher	Cat# FB1000
Multipurpose rotator	Thermo Scientific	Model#2314

**Experimental models: organisms/strains**		

*Schmidtea mediterranea* CIW4 clonal strain	n/a	n/a

## Materials and equipment

**Table:** 10× Running buffer

Reagent	Final concentration or percentage	Amount
Tris base	250 mM	30.3 g
Glycine	1.92 M	144 g
SDS	1%	10 g
MilliQ water	n/a	Up to 1 L
**Total**	**n/a**	**1 L**

**Table:** 6× Laemmli buffer, pH 6.8

Reagent	Final concentration or percentage	Amount
Tris base	375 mM	1.47 g
SDS	6%	1.5 g
Glycerol	4.8%	1.2 mL
2-Mercaptoethanol	9%	1 mL
Bromophenol blue	0.03%	1 mL
MilliQ water	n/a	Up to 25 mL
**Total**	**n/a**	**25 mL**

**CRITICAL:** 2-Mercaptoethanol is toxic and should always be used with adequate ventilation, preferably in a fume hood. Follow the safety data sheet when handling 2-mercaptoethanol.

***Note:*** Combine Tris base, SDS, and glycerol and heat to 40°C. Once in solution, 2-mercaptoethanol and Bromophenol blue should be added. Solution needs to be adjusted to a pH of 6.8 using HCl, and brought up to a final volume of 25 mL with MilliQ water.

**Table:** 10× Transfer buffer

Reagent	Final concentration	Amount
Tris base	250 mM	30.3 g
Glycine	1.92 M	144 g
MilliQ water	n/a	Up to 1 L
**Total**	**n/a**	**1 L**

**Table:** 10× PBS, pH 7.0

Reagent	Amount
NaCl	80 g
KCl	2 g
Na_2_HPO_4_	14.4 g
KH_2_PO_4_	2.4 g
MilliQ water	Up to 1 L
**Total**	**1 L**

**Table:** 1× TBST

Reagent	Final concentration or percentage	Amount
Tris base (1 M, pH 7.4)	20 mM	50 mL
NaCl (5 M)	140 mM	30 mL
Tween-20	0.1%	1 mL
MilliQ water	n/a	919 mL
**Total**	**n/a**	**1 L**

**Table:** 1× TBST/SDS

Reagent	Final concentration or percentage	Amount
Tris base (1 M, pH 7.4)	20 mM	12.5 mL
NaCl (5 M)	140 mM	7.5 mL
Tween-20	0.2%	0.5 mL
SDS (20%)	0.01%	125 μL
MilliQ water	n/a	229.375 mL
**Total**	**n/a**	**250 mL**

**Table:** 15% acrylamide gel solution

Reagent	Final concentration or percentage	Amount
MilliQ water	n/a	1.250 mL
30% acrylamide	15%	2.75 mL
Tris (1.5 M, pH 8.8)	375 mM	1.375 mL
SDS (10%)	1%	55 μL
APS (10%)	1.18%	65 μL
TEMED	0.09%	5 μL
**Total**	**n/a**	**5.5 mL**

**CRITICAL:** Acrylamide and TEMED are toxic and should always be used with adequate ventilation, preferably in a fume hood. Follow the safety data sheet when handling acrylamide and TEMED.

***Note:*** APS and TEMED are polymerizing agents and should be added last. The percentage of acrylamide can be adjusted (replaced with MilliQ water) accordingly depending on the size of the protein of interest.

**Table:** Acrylamide stack solution

Reagent	Final concentration or percentage	Amount
MilliQ water	n/a	1.37 mL
30% acrylamide	5%	335 μL
Tris (1.0 M, pH 6.8)	125 mM	250 μL
SDS (10%)	1.0%	20 μL
APS (10%)	1.0%	20 μL
TEMED	0.25%	5 μL
**Total**	**n/a**	**2 mL**

**CRITICAL:** Acrylamide and TEMED are toxic and should always be used with adequate ventilation, preferably in a fume hood. Follow the safety data sheet when handling acrylamide and TEMED.

***Note:*** Stacking solution polymerizes within 5–10 min, and should be made only when ready. APS and TEMED are polymerizing agents and should be added last.

**Table:** 1× RIPA buffer

Reagent	Final concentration or percentage	Amount
MilliQ water	n/a	720 μL
10× RIPA buffer	1×	100 μL
DTT (100 mM)	1 mM	10 μL
PMSF (100 mM)	1 mM	10 μL
Protease Inhibitor Cocktail	15%	150 μL
Phosphatase Inhibitor Cocktail	1%	10 μL∗
**Total**	**n/a**	**1 mL**

***Note:*** Components for making a 1× RIPA buffer should be added in the order listed above. Protease inhibitors (PMSF and Protease Inhibitor Cocktail) should be added last and only when samples are ready. ∗ Phosphatase Inhibitor Cocktail should be used only if the protein of interest can be phosphorylated. If Phosphatase Inhibitor Cocktail is added, this should be added immediately before samples are ready.

## Step-by-step method details

### Acrylamide gel casting: day 1

**Timing: 0.5–1 h**

Casting acrylamide gels by hand is a cheaper alternative to purchasing pre-casted gels. The benefit to casting your own gels is the flexibility to change the percentage of acrylamide depending on the size of the proteins you are interested in. The process of casting gels is straight forward and can easily be done if the gel casting equipment is in relatively good condition.

1. First, ensure that gel casting equipment is clean, wash off with DI water if necessary. Assemble glass gel plates (spacer plate and short plate) together by locking them firmly in the casting frame.

***Note:*** Make sure glass plates align on the bottom and glass is not severely chipped, this will prevent leaking.

**Figure 1:**
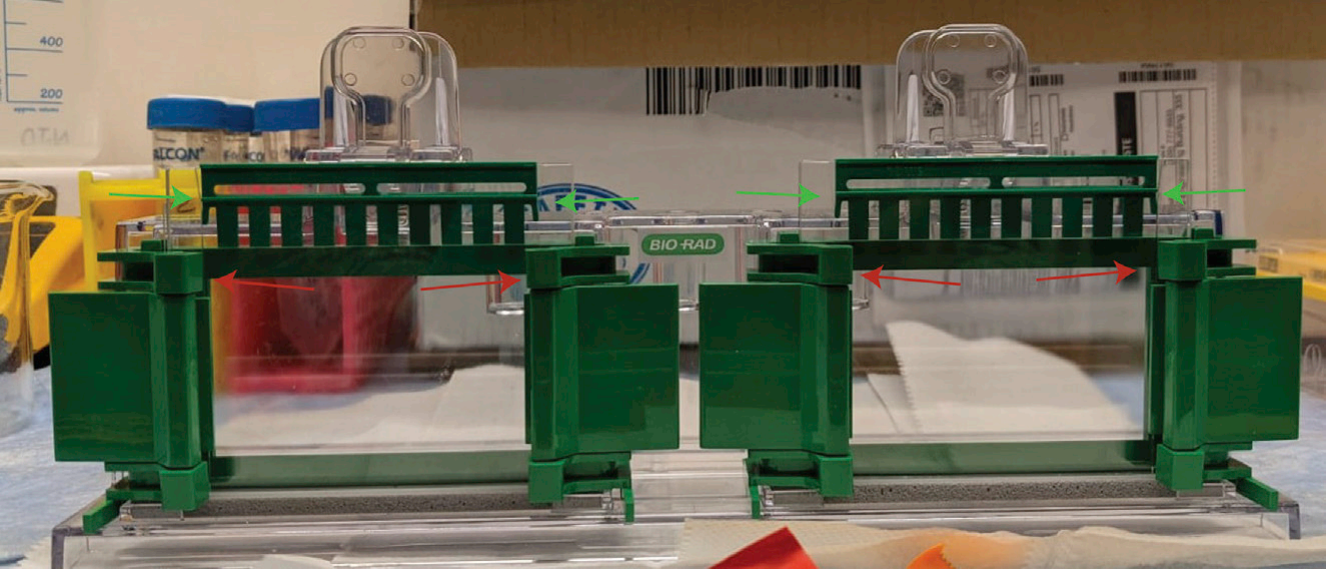
Gel casting system Image displaying the acrylamide gel casting system. The red arrows indicate the fill line for the acrylamide gel preparation and the green arrows indicate the top of the of the short plate.

2. Set up gel casting module in the fume hood by first placing rubber gaskets in the casting stand, then clamping in the casting frame with glass gel plates (Figure 1).

**CRITICAL:** To avoid excessive cleaning, place paper towels under the gel casting module in case of leakage. Casting acrylamide gels uses toxic chemicals and all steps should be performed in the fume hood.

3. Prepare the acrylamide gel solution in a 15 mL conical tube. Add the MilliQ water, 30% acrylamide, 1.5 M Tris (pH 8.8), 10% SDS, 10% APS, and TEMED in the order listed. Refer to Materials and equipment for the acrylamide gel recipe.
4. Vortex acrylamide gel solution for 5–10 s. Using a serological pipette, transfer acrylamide gel solution between casting plates, stopping ∼2–3 cm from the top of the short plate.

***Note:*** It is important not to go higher than 2–3 cm from the top. You will need sufficient room to add the acrylamide stack solution and the comb (Figure 1).

5. Add 500 μL of Isopropanol on top of acrylamide gel solution, between the glass plates, and evenly distribute to remove all air bubbles. Gels will require 15–45 min to solidify.

***Note:*** If gel casting begins leaking from the bottom, carefully transfer spilled solution back into the 15 mL conical tube used to prepare the mix. Let the gel completely solidify before removing the gel mixture, which will prevent further spilling over the casting module. Once the gel has solidified, carefully use the gel releaser to transfer the gel into the proper disposal container. Glass plates can be cleaned first using Isopropanol, followed by DI water. Ensure that both the rubber gasket and glass plates are fully dried before going back to step 1.

6. Once the gel has completely solidified, carefully remove all Isopropanol. This can be done with a paper towel or piece of blotting filter paper.
7. Prepare the acrylamide stack solution in a 15 mL conical tube. Add the MilliQ water, 30% acrylamide, 1.5 M Tris (pH 8.8), 10% SDS, 10% APS, and TEMED in the order listed. Refer to Materials and equipment for the acrylamide stack solution recipe.
8. Vortex acrylamide stack solution for 5–10 s. Use a transfer pipette to transfer the acrylamide stack solution between the casting plates. Fill the plates to the top of the short plate, ensuring there are no air bubbles remaining on top.
9. Place the comb into the acrylamide stack solution and let the acrylamide stack solution solidify ∼5–10 min.

***Note:*** Transfer any spilled acrylamide stacking solution back into the 15 mL conical tube used to prepare the mix. You do not want the spilled solution to solidify on the outside of the gel casing. Remaining acrylamide stack solution should be properly disposed; this includes toxic chemicals.

10. Once the acrylamide stack has solidified, carefully release gel from the casting and place into a bag containing ∼50–100 mL of 1× Running buffer. Refer to Materials and equipment for the 1× Running buffer recipe.
11. Place the gel flat in a 4°C fridge, submerged in the 1× Running buffer. The gel needs to set for at least 8 h and not exceeding 7 days before being used.

***Note:*** It is best to clean off gel casting components immediately. Use Isopropanol followed by DI water to prevent buildup of solidified acrylamide solutions.

### Protein extractions: day 2

**Timing: 1.5–3 h**

The precision of the protein extractions is the most critical part of this protocol. The quality of the protein and the correct measurement of protein levels in samples will ultimately determine the quality of the western blotting. For beginners, try minimizing the number of samples to reduce error.

12. Prepare the 1× RIPA buffer by adding MilliQ water, 10× RIPA buffer, 100 mM DTT, 100 mM PMSF, Protease Inhibitor Cocktail, and if needed Phosphatase Inhibitor Cocktail, into a 1.5 mL centrifuge tube and store on ice. Refer to Materials and equipment for the 1× RIPA buffer recipe.

***Note:*** 100 mM PMSF will need to be rocked at room temperature or heated to 37°C to go into solution. 100 mM DTT, 10× RIPA buffer, and Protease Inhibitor Cocktail can thaw on ice. Proteases (PMSF and Protease Inhibitor Cocktail) should be added last to the 1× RIPA buffer solution. If protein of interest is phosphorylated, add the Phosphatase Inhibitor Cocktail last. Depending on the number of samples, volumes of 1× RIPA buffer can be adjusted accordingly.

13. Use a transfer pipette to place 10–30 starving animals into labeled 1.5 mL centrifuge tubes. Leave ∼300 μL of water behind.

***Note:*** Having more remaining water will slow down the process of removing water between samples. Having not enough water will cause animals to stress and secrete excess mucus.

14. Quickly remove remaining water and add 150–200 μL of 1× RIPA buffer to the sample. Keep sample on ice.
  a. Repeat step 13 for each sample.

***Note:*** The animals being referenced here are the asexual strain *Schmidtea mediterranea.* Please take into consideration the length of the animals when performing this on either *Schmidtea mediterranea* or other planarian species, as this factor may influence the amount of 1× RIPA buffer being required for each sample. For fewer or smaller animals (<3 mm length), use ∼100–150 μL of 1× RIPA buffer (see TroubleshootingProblem 1 for details). For more or larger animals (>8 mm length), use∼150–300 μL of 1× RIPA buffer.

15. Using a motorized pestle, homogenize each sample for ∼10–20 s on ice.

***Note:*** To avoid contamination, use a new sterile pestle for each sample and discard after use.

16. Incubate samples on ice for 45 min.
17. Using a pre-cooled centrifuge, spin the samples at 20,817 × *g* for 20 min at 4°C.
18. Carefully remove the supernatant from the sample and transfer into a clean, labeled 1.5 mL centrifuge tube.

***Note:*** Avoid removing supernatant too close to the pellet, this may result in debris contaminating samples.

19. Set up the Bradford Assay.
  a. Label 0.2 mL PCR tubes for triplicates/sample (ex. Sample 1A, Sample 1B, Sample 1C, etc.).
  b. Add 95 μL of MilliQ water to each 0.2 mL tube.
  c. Add 5 μL of sample protein extract to each of the corresponding labeled tubes.
  d. Briefly vortex samples and placed on ice.
  e. Add 20 μL of the BSA standard (0–10 μg/μL) into wells of the 96 well plate in triplicates (3 wells/standard).
  f. Add 20 μL of the sample extraction into wells of the 96 well plate in triplicates (3 wells/0.2 mL sample tube).
  g. Use a multichannel pipette to add 200 μL of Bradford Reagent into each well. Carefully mix each well, while avoiding air bubbles.

***Note:*** The set up above would provide corresponding values between BSA standards and sample protein extraction. For example, the BSA standard 1 μg/μL would have a total of 20 μL/well and hence 20 μg of total protein in that well. The sample protein extract is diluted 1:20 (5 μL/95 μL) and 20 μL would be added to that well. If the sample protein extract had the sample corresponding value as the 1 μg/μL BSA standard, the sample protein extract would contain 1 μg/μL of total protein.

20. Use a plate reader with a 595 nm wavelength to read the 96 well plate.
  a. Include the transformations in the software: Blank correction (all wells corrected by a common blank group), standard curve fit (linear regression with standard), %CV (calculates %CV of concentrations).
21. Export the 96 well plate reads as an excel file.
22. Use Excel to average sample protein extracts between the triplicates (see Table1 for an example).
  a. Calculate 50 μg of total protein (in μL) for each sample.
  b. 1× Laemmli buffer (in μL) needs to be included for each sample, using the same amount across the samples.
  c. Use MilliQ water to create equal volumes across the samples.

**Table 1:** Sample preparation for western blotting

	Control	TRAF1(RNAi) day 5	TRAF1(RNAi) day 7	TRAF1(RNAi) day 9	TRAF2(RNAi) day 5	TRAF2(RNAi) day 7	TRAF2(RNAi) day 9
Sample A	8.80	8.11	8.39	8.62	8.29	8.96	8.86
Sample B	8.82	7.98	8.52	8.93	8.41	8.75	8.77
Sample C	8.91	8.09	8.26	8.85	8.56	8.89	9.00
Average (μg/μL)	8.84	8.06	8.39	8.80	8.42	8.86	8.87
50 μg (μL)	5.65	6.20	5.96	5.68	5.94	5.64	5.63
Water (μL)	6.85	6.30	6.54	6.82	6.56	6.86	6.87
6× Laemmli buffer (μL)	2.50	2.50	2.50	2.50	2.50	2.50	2.50
Total volume (μL)	15.00	15.00	15.00	15.00	15.00	15.00	15.00

***Note:*** You want loading volumes to be identical across samples. Once the volume of loading is determined, you can calculate the amount of 6× Laemmli buffer needed to make the samples 1×, and the amount of MilliQ water that needs to be added to each sample. This means that MilliQ water will be added variably to samples. Aim to load wells with less than 20 μL of sample, so there will be minimal risk of samples leaking into neighboring wells. Loading volumes can be altered depending on the size of the gel (see TroubleshootingProblem 1 for details).

### Western blotting: days 2–4

**Timing: 2–3 days**

The results of western blotting depend heavily on the transfer of proteins from the acrylamide gel to the membrane.

23. Prepare 50 μg samples for loading in 1.5 mL centrifuge tubes, from calculations in step 22. Mix samples by gently pipetting up and down between solutions.
24. Heat samples at 94°C for 10 min.

***Note:*** Depending on the size, total number, or strain of planarians, longer boiling times may be required, as mucus content may interfere with SDS-PAGE results.

25. Allow samples to cool down to room temperature.
26. Quickly spin down samples using a centrifuge.

***Note:*** Do not over-centrifuge samples, otherwise you will produce a pellet in the sample.

27. Set up gels in the buffer tank.
  a. Remove gel(s) from the 4°C fridge and quickly wash with DI water.
  b. Carefully remove the comb and rinse the wells with DI water, to remove debris.
  c. Assemble the gel running module by placing gels in the correct orientation, across from one another (short plates facing inward).
  d. Fill the running module with 1× Running buffer and buffer tank to the fill line with 1× Running buffer.

***Note:*** If only one gel is being run, place a plastic buffer dam in place of the second gel.

28. Load the acrylamide gel(s).
  a. Load each sample into the wells.
  b. Load 2.5 μL of protein ladder to the bordering wells.
29. Run gel(s) for 1.5–2 h using a voltage ∼125 V.

***Note:*** Cut off the power supply when the dye front reaches the very bottom of the gel. This may occur before or after 1.5–2 h.

30. Activate the PVDF membrane
  a. Submerge the membrane in 100% methanol while rocking at room temperature for 1 min.
  b. Transfer the membrane and submerge into 1× PBS while rocking at room temperature for 1 min.
  c. Transfer the membrane and submerge into 1× Transfer buffer and rock at room temperature for 10 min.

***Note:*** Try to flip the membrane over halfway through each of the solution incubations listed above.

31. Prepare the transfer sandwich.
  a. Cut out blotting filter paper to be slightly larger than the size of the membrane, and soak in 1× Transfer buffer.
  b. Soak sponges in 1× Transfer buffer.
  c. Carefully pry open the gel casing, remove the stacking portion and bottom of the gel (dye front). Place the gel in 1× Transfer buffer.
  d. Open the transfer cassette, placing the black side in front of you. Do this on a surface that will keep liquid from spilling.
  e. To the black side of the transfer cassette, place a soaked sponge, followed by a soaked piece of blotting filter paper.
  f. Carefully place the membrane on top of the filter paper, followed by the gel, with the low molecular weight markers facing out and the high molecular weight markers facing the clear side of the transfer cassette. Keep in mind the orientation of the gel, in terms of samples.
  g. Carefully place a soaked blotting filter paper on top of the gel, followed by a soaked sponge.
  h. Gripping the left and right side of the sandwich, flip the sandwich so that the gel is now on the bottom with the membrane on top, and the low molecular weight marker is now facing the inside of the transfer cassette.
  i. Add a small amount of 1× Transfer buffer on top of the sandwich and remove air bubbles by using a roller.
  j. Carefully close the transfer cassette and place it inside the transfer module, with the black portion of the transfer cassette facing the black portion of the module.
32. Set up the Transfer.
  a. Fill a large ice bucket with ice and place the buffer tank in the center.
  b. Add the transfer module and frozen ice pack into the buffer tank.
  c. Add 1× Transfer buffer into the buffer tank, until the transfer module is submerged.
  d. Place the lid on the buffer tank and cover the buffer tank with additional ice.
  e. Run the transfer for 1 h using a voltage ∼55 V.
33. Remove the transfer cassette, and carefully remove the membrane from the sandwich. Place the membrane into a western blot incubation box containing 5 mL 1× TBST, with the protein side of the membrane facing up.
34. Prepare 10 mL primary blocking solution (1× TBST, 5% BSA w/v).

***Note:*** It is important to not use nonfat dry milk powder for detecting phosphorylated proteins. Milk contains phosphorylated proteins, such as casein which can produce increased background upon detection.

35. Remove 1× TBST from the membrane and replace with 5 mL of primary blocking solution.
36. Incubate membrane for 1 h, rocking at room temperature.
37. Prepare primary antibody.
  a. Dilute Anti-Caspase-3, 1:500 into 5 mL primary blocking solution.

***Note:*** Determination of the appropriate dilution ratio will need to be established when testing primary antibodies for the first time (see TroubleshootingProblem 2 for details).

38. Remove primary blocking solution from the membrane and replace with 5 mL of primary antibody.
39. Incubate primary antibody for 16 h, rocking at 4°C.

**Pause point:** continue with further incubation and washes the next day.

***Alternatives:*** Instead of incubating the primary antibody for 16 h at 4°C, 4 h at room temperature will suffice.

40. Remove the primary antibody and replace with 5 mL of 1× TBST.
41. Perform (4×) 20-min washes with 5 mL of 1× TBST, while rocking the membrane at room temperature.
42. Prepare secondary antibody.
  a. Dilute Anti-HRP, Goat-Anti-Rabbit, 1: 4,000 in 5 mL 1× TBST-SDS, 5% nonfat dry milk powder w/v).
43. Remove 1× TBST and replace with secondary antibody.
44. Incubate secondary antibody for 1 h, rocking at room temperature.
45. Remove secondary antibody and replaced with 5 mL of 1× TBST.
46. Perform (4×) 20-min washes with 5 mL of 1× TBST, while rocking the membrane at room temperature.
47. Remove 1× TBST and replace with 5 mL of 1× PBS.
48. Perform (2×) 5-min washes with 5 mL of 1× PBS, while rocking the membrane at room temperature.
49. Chemiluminescence detection.
  a. Place sample tray for chemiluminescence detection on to the ChemiDoc imager.
  b. Place a clear piece of plastic onto the chemiluminescence detection tray.
  c. Carefully place the membrane protein side up, on top of the clear plastic.
  d. Using a paper towel, carefully remove remaining liquid.
  e. Add 250 μL of HRP chemiluminescence substrate on top of the membrane.
  f. Carefully place another piece of plastic on top of the membrane, spreading the substrate to remove air bubbles.
  g. Image blot under chemiluminescence, using the Optimal Auto-exposure setting with higher resolution for detection of antibody bound protein.
  h. Image blot under colorimetric, using the Optimal Auto-exposure setting with higher resolution for detection of molecular weight marker.
  i. On the ChemiDoc, merge the files together.

***Note:*** A plastic zip-lock bag with the all but one side removed works excellent for chemiluminescence detection.

50. Carefully transfer the membrane back into the western blot incubation box.
51. Perform (2×) 5-min washes with 5 mL of 1× PBS, while rocking the membrane at room temperature.
52. Remove 1× PBS and replace with 5 mL of western blot stripping buffer.
53. Incubate western blot stripping buffer for 15 min, while rocking the membrane at room temperature.
54. Remove western blot stripping buffer and replace with 5 mL 1× PBS.
55. Perform (4×) 5-min washes with 5 mL of 1× PBS, while rocking the membrane at room temperature.
56. Replace 1× PBS with 5 mL of 1× TBST.
57. Repeat steps 34–36.
58. Prepare primary antibody.
  a. Dilute Anti-Actin, 1: 3,000 into 5 mL primary blocking solution.
59. Repeat steps 39–41.
60. Prepare secondary antibody.
  a. Dilute Anti-HRP, Goat-Anti-Mouse, 1: 10,000 in 5 mL 1× TBST-SDS, 5% nonfat dry milk powder w/v).
61. Repeat steps 43–49.

***Note:*** Internal control band intensities should be relatively similar between all sample and not over saturated. If the band intensities greatly differ, this may indicate unequal protein loading. Over saturation of bands may lead to inaccurate quantification (see TroubleshootingProblem 3 for details).

## Expected outcomes

The protein extraction protocol is designed to obtain the maximum protein concentration while having enough protein to measure and assemble samples for western blotting. Using averages from 9 individual wells/sample, protein concentrations should be fairly accurate. The total protein concentrations are expected to range from 5–10 μg/μL. This should enable loading of less than 20 μL of sample into the wells of the acrylamide gel.

**Figure 2:**
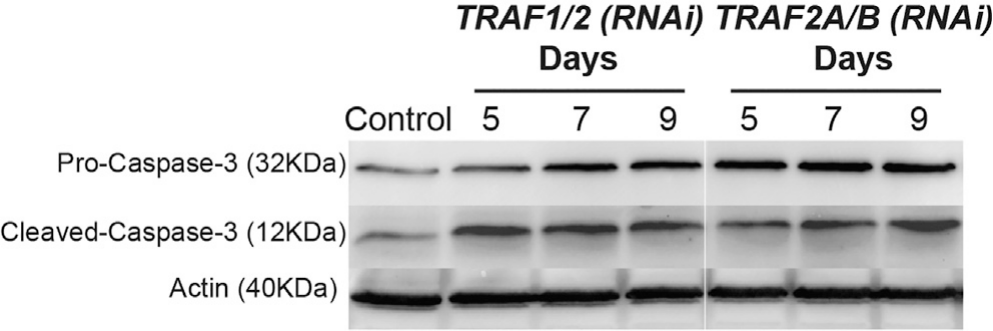
Antibody detection on membrane Western blot membrane detecting levels of Pro-Caspase-3, Cleaved-Caspase-3, and Actin. These data are from the original Figure 2A in Ziman et al. (2020a).

Equal loading of 50 μg of protein should be evident by the band intensities of the internal control (anti-actin). Some anti-actin band intensities may vary. However, due to the fact that we are using anti-actin to normalize anti-caspase-3, this will be okay (Ziman et al., 2020a) (Figure 2). No data will be lost due to slightly more or less protein being present.

## Quantification and statistical analysis

1. Open images using the Bio-Rad software, Image Lab 6.0.1.
2. Export images.
  a. Under file, select export, and export for publication, using a 600 dpi publishing resolution.
  b. Save image as a TIFF Image.

3. Use ImageJ for quantifying band intensities.
  a. Import TIFF image into ImageJ.
  b. Use the rectangle tool to draw a box that fits the size of a single band of interest, without overlapping other bands.
  c. Select the first band by pressing crtl and 1 (PC) or command and 1 (MAC).
  d. Drag the first box over the second band and press ctrl and 2 (PC) or command and 2 (MAC).
  e. Repeat with (d) for each of the bands.
  f. Plot selected lanes by pressing ctrl and 3 (PC) or command and 3 (MAC).
  g. Use the “Straight line” tool to add a line across the bottom of the peaks, so that you can measure the area under the curve.
    ***Note:*** Make sure the line overlaps with the outside of the curve.
  h. Use the “wand” tool to measure the area under the curve by clicking inside peak.

4. Using Excel, record the band intensities for both anti-caspase-3 and anti-actin. Normalize anti-caspase-3 activity to anti-actin, by diving the area under the curve value of anti-caspase-3 by the area under the curve value for anti-actin. Data can be represented in fold change back dividing the normalized sample data by the normalized control data.

## Limitations

This protocol is for measuring proteins within the cytoplasmic fraction of the cell. This protocol has been tested with a handful of different antibodies but may not work with all antibodies.

## Troubleshooting

### Problem 1

Low levels of total protein per sample (steps 14, 22).

### Potential solution

In this protocol we recommend loading 50 μg of total protein per sample, to obtain robust results. The number/size of animals and the volume of 1× RIPA buffer will determine the total protein being extracted. When using fewer/smaller animals, lowering the volume of 1× RIPA buffer may be necessary. In this protocol we recommend using ∼100–150 μL of 1× RIPA buffer for smaller/fewer animals. You may lower this volume further, just take into consideration that ∼5–15 μL should be used for the Bradford Assay, sample preparation will most likely require ∼5–15 μL, and that some volume will be lost in order to avoid contamination of debris. While we recommend using ∼100–150 μL of 1× RIPA buffer, this may be cut in half under given circumstances.

We also recommend that the volume being loaded is less than 20 μL/ well. However, this volume can be increased. Depending on the size of the gel casing (0.75 mm, 1.0 mm, 1.5 mm) larger gaps between the glass plates can increase the total volume of sample. For example, if you require 10 wells on the gel, the maximum sample volume per well is 33 μL, 44 μL, and 66 μL for 0.75 mm, 1.0 mm, 1.5 mm wells, respectively.

In the situation in which where you are unable to change the sample volume and fall short of 50 μg, this can be managed. Increasing the primary and/or secondary antibody concentrations can enhance the amount of signal being produced.

### Problem 2

Determine the dilution ratio for primary antibodies (step 37).

### Potential solution

It is important to establish the dilution ratio for the primary antibody of interest, to reduce background on western blotting. For antibodies that have not been previously used for western blotting, start by using the same dilution factor that has been established for immunohistochemistry, and adjust accordingly. For antibodies that have never been tested, it is recommended that a protein concentration gradient ranging from 10–50 μg be used, diluting the primary antibody according to manufacturer’s recommendation for western blotting or immunohistochemistry (if western blotting is not provided). In the situation where there is no manufacturer’s recommendation for dilution, start by performing a range of dilutions between 1:250–1: 10,000. It is important to take the total amount of protein being loaded into consideration when determining the primary antibody concentration. Ensure that the amount of protein does not oversaturate the intensity of the loading control antibody. For example, if 60 μg and 100 μg of protein oversaturates the band intensity of the loading control, this loading control will not reflect an accurate overall quantification of the primary antibody of interest. If the loading control antibody has been established to work under certain protein concentrations, do not exceed this protein concentration when determining the dilution ratio for the primary antibody of interest.

### Problem 3

Uneven or over saturation of internal control band intensity. The internal control is critical for quantifying the relative protein of interest levels (step 61).

### Potential solution

The best way to ensure that the protein loading and hence the internal band intensities are relatively even is consistency between samples. This means that the number/size of animals are fairly similar across samples, as well as the sample quantity of 1× RIPA buffer being added to each sample. Therefore, the total protein between samples should remain relatively the same, in case the BSA standard is not completely linear.

In the situation where the band intensity appears over saturated, this may be a result of over exposure when imaging. To circumvent this problem, try imaging with the manual exposure setting, and reducing the time length of the exposure. If very low exposures still produce over saturation of internal control band intensities, consider diluting primary and/or secondary antibody concentrations. Another possibility may be the internal control antibody itself. If the required total protein loading concentration is too high for the internal control antibody and results in saturation after modifying the exposure time and antibody concentrations, consider using another internal control antibody that will not produce the same effect. As an alternative to anti-actin, beta-tubulin has shown to be an effective internal control antibody (Ziman et al., 2020b).

## Resource availability

### Lead contact

Further information and requests for resources and reagents should be directed to and will be fulfilled by the Lead Contact, Néstor J. Oviedo (noviedo2@ucmerced.edu).

### Materials availability

This study did not generate new unique reagents.

### Data and code availability

This study did not generate/analyze [datasets/code].
